# Clinical Applications of Photofunctionalization on Dental Implant Surfaces: A Narrative Review

**DOI:** 10.3390/jcm11195823

**Published:** 2022-09-30

**Authors:** Li-Ching Chang

**Affiliations:** 1 Department of Dentistry, Chang Gung Memorial Hospital, Chiayi 61363, Taiwan; liching@ms39.hinet.net; 2 Institute of Nursing and Department of Nursing, Chang Gung University of Science and Technology, Chiayi 61363, Taiwan

**Keywords:** ultraviolet, dental implant, osseointegration, bone–implant interface

## Abstract

Dental implant therapy is a common clinical procedure for the restoration of missing teeth. Many methods have been used to promote osseointegration for successful implant therapy, including photofunctionalization (PhF), which is defined as the modification of titanium surfaces after ultraviolet treatment. It includes the alteration of the physicochemical properties and the enhancement of biological capabilities, which can alter the surface wettability and eliminate hydrocarbons from the implant surface by a biological aging process. PhF can also enhance cellular migration, attachment, and proliferation, thereby promoting osseointegration and coronal soft tissue seal. However, PhF did not overcome the dental implant challenge of oral cancer cases. It is necessary to have more clinical trials focused on complex implant cases and non-dental fields in the future.

## 1. Introduction

Dental implant therapy is a common clinical procedure for the restoration of missing teeth. Further, the surface wettability of dental implant might affect osseointegration [1]. It was first found in 1997 that ultraviolet (UV) irradiation could induce superhydrophilicity in titanium dioxide (TiO_2_) because the surface oxygen vacancies at bridging sites result in the conversion of relevant Ti4^+^ sites to Ti3^+^ sites for more water adsorption [2,3]. In addition to applications in antifogging and self-cleaning materials, the concept was also used in dental implant treatment [3,4,5].

Photofunctionalization (PhF), first described in 2009, is defined as an overall phenomenon of titanium surface modification after UV treatment, including the alteration of physicochemical properties and the enhancement of biological capabilities [4,5]. UV radiation is categorized into UVA (wavelength λ = 320–400 nm), UVB (λ = 280–320 nm), and UVC (λ = 200–280 nm). The effects of PhF include the modification of the implant surface from hydrophobic to super-hydrophilic and the reversal of its biological aging [1,6,7,8]. Moreover, PhF could also decrease the amount of bacterial attachment/accumulation and maintain the antimicrobial surface in vitro [9,10,11].

The clinical application of PhF was first reported in 2013 and included seven implants in four implant complex cases utilizing PhF before implant placement [12]. Herein, we introduce the effects of PhF and its recent clinical applications. The aim of this article was to focus on clinical studies of PhF from January 2013 to June 2022. The digitally searched papers from PubMed used “photofunction” and “dental implant” as key words. After abstract review, there were two double-blind clinical trial studies, three prospective studies, four retrospective studies, and one case series in the present paper [12,13,14,15,16,17,18,19,20,21]. Furthermore, PhF applied in the spine surgery of 13 patients was included [22]. Except for some pre-clinical review papers on in vitro or animal studies, to my best knowledge, this paper is the first review focused on the clinical application of photofuctionalization [4,23,24,25].

## 2. In Vitro Studies

A brief summary of in vitro studies is shown in Table 1.

The water contact angle on the titanium surface was reduced to 0.5° after PhF; thus, the surface was changed from hydrophobic to super-hydrophilic [1,41]. The titanium implant surfaces had harmful and time-dependent degradation due to carbon contamination (hydrocarbons), which was defined as “the biological aging of titanium” [6,27,28]. Roy reported that titanium implant surfaces, as little as 4 weeks from production, are contaminated by atmospheric hydrocarbons [26]. The 4-week-old titanium implants required more osseointegration time than the newly prepared titanium implants by two-fold. The bone-to-implant contact (BIC) percentage on the 4-week-old surfaces was less than the BIC on the new surfaces (60% vs. 90%). Additionally, only 20% to 50% of the levels of recruitment, attachment, and proliferation of osteoblasts showed on the 4-week-old surface when compared with new surfaces [3]. The PhF could reduce the concentration of surface hydrocarbons on different implants by three- to four-fold, thus improving biologic results [26,27,29] and no change in the topography of implant surfaces [7,27,29]. Furthermore, PhF could increase the oxygen concentration of the zirconia implant surfaces and decrease carbon concentration [30]. There was increased protein adsorption, as well as the improved migration, attachment, and proliferation of osteoblasts on photofunctionalized surfaces in vitro [12,31,32]. In addition, UV treatment could restore the reduction in the bioactivity of titanium implants, which was adverse effect of temperature deviations when handling titanium materials [35]. With the exception of osteoblasts, the attachment of gingival fibroblasts or epithelial cells was also enhanced on UV-treated titanium and the zirconia abutment surface, which could enhance the soft tissue seal of the peri-implant interface [36,38,39,40]. In addition, the UV-photofunctionalization of instruments could prevent infection by restricting the growth of oral bacteria and biofilm and suppressing the proinflammatory gene expression of IL-1β [9,10,11,37].Therefore, PhF may be a useful and easy adjunctive method to improve osseointegration by utilizing a combination of these advantages.

The average BIC is reported to be 45%, which is lower than the ideal 100% [12]. PhF could result in a super-hydrophilic implant surface, reversed the biological degradation of the implant surface, and optimized surface electrostatic charges [3,4,12]. Thus, PhF improves BIC by up to 98.2% and promotes osseointegration [3,4,5]. The bone morphogenesis around UV-irradiated titanium surfaces was known as “superosseointegration” [3,5,6]. Additionally, reduced stress on the surrounding tissues with improved stress distribution was found on UV-treated implants when compared with UV-untreated implants using a three-dimensional finite element analysis model, especially under vertical loading [31,32].

## 3. Preclinical Animal Studies

Additional hydrocarbons on titanium implant/instrument surfaces decreases bone-binding ability by aging [22]. However, PhF could promote osseointegration by reducing hydrocarbons [22,33]. Shen reported that UV PhF eliminated hydrocarbon contamination with resultant enhanced BIC and osseointegration in a rabbit model [42]. This increase in BIC was found in rat, rabbit, and dog models [1,43,44,45,46,47,48,49,50]. However, there were no significant differences in BIC and implant stability quotient (ISQ) between the UV treatment and control group 9 months after implant placement in the jaw bone of mini-pigs [51]. Except for titanium, the BIC was also enhanced in zirconia-based material by 3 to 7-fold in smooth surfaces and by 1.4 to 1.7-fold in rough surfaces [43].

The osseointegration of custom-made or commercial dental implants was accelerated by PhF in different animal models; in other words, an earlier osseointegration was achieved by UV treatment [5,8,34,49,51]. The biological enhancing effect remained even after 12 weeks of healing in a rabbit model [52]. A 2.2~2.3-fold increase in the strength of osseointegration was found in normal rats, and the genetically modified rats (close to human diabetes) showed a 1.8 to 3-fold increase after using UV treatment (TheraBeam Affiny device) for 15 min [53,54]. The strengtth of osseointegration in the aged rats was enhanced by 40% after UV treatment (TheraBeam SuperOsseo device) for 12 min [55]. Moreover, bone-implant integration after PhF was 80% higher than that of the control titanium implants in ovariectomized rats (close to osteoporosis) [41].

When implants were subjected to constant lateral force during healing, an increased implant success rate was seen in photofunctionalized surface group as compared with the control group (100% vs. 28.6% respectively) in a rat study [56]. In addition, PhF increased the orthodontic mini-screw’s resistance against tipping force by 1.5~1.7-fold and resulted in less displacement under a lateral tipping force in rats [57,58]. Therefore, it is impossible to gain more anchorage of orthodontic mini-screws clinically by using UV treatment. Except for the PhF with commercial UV machines, the use of a bacterial UV bench lamp (wavelength of 254 nm) for 48 h also increased the volume of cortical-like tissue in the coronal region in a rabbit study [9]. The early osseointegration of aged titanium implants in a dog model could be enhanced by ultraviolet-C light photofunctionalization. However, the effect was independent on UVC exposure within a range from ten minutes to one hour [59]. Therefore, the UV treatment time using a bench lamp is too long for clinical use when compared with UV machines that require 12 or 15 min.

Finally, a summary of the preclinical animal studies is shown in Table 2.

## 4. Clinical Studies

Ten clinical papers were associated with dental implant therapy using photofunctionalization. Most papers (7/10) were from the Ogawa study group in Japan [12,13,14,15,16,17,18]. These papers are summarized in Table 3.

The first clinical report in 2013 by Funato et al. revealed that the ISQs for seven implants placed in compromised bone after PhF for 15 min increased from a range of 48~75 at placement to 68~81 at loading [12]. Funato’s further retrospective study showed that PhF could shorten healing time from 6.6 months to 3.2 months before loading when compared with the control group; however, the implant survival rates of both groups were similar [13]. This means that PhF would enhance the dental implant osseointegration speed index (OSI) [12,13]. In addition, the same result was also found in Suzuki’s prospective research [14]. Moreover, the implant stability dip was eliminated by PhF; especially implants with less primary stability could obtain more ISQs gain using a TheraBeam Affiny machine [12,14,15]. UV treatment has chemical and biological effects on the osseous–implant interface, and PhF for as little as 15 min could enhance BIC and promote osseointegration [64].

In comparison with regular cases, PhF was more effective in complex cases, including cases with ridge augmentation, sinus elevation, and immediate implant [16]. PhF is a stronger determinant of implant stability than the other patient-related and implant-site-related factors [16]; thus, PhF results in a lower early failure rate than that in the non-UV treatment group (1.3% vs. 4.3%) in a large retrospective study [17]. However, PhF still did not overcome the pathophysiological condition of cancer-related complex cases with bone resection, segmental defect, or radiation, in which the implant survival rate was only 22.2% [18].

In addition to Japan’s studies, which used a TheraBeam Affiny (Ushio Inc., Tokyo, Japan) for 15 min, a recent Korean clinical trial also used the same UV machine [29]. The UV light of the TheraBeam Affiny was delivered as a mixture of spectra via a single source UV lamp at λ = 360 nm and λ = 250 nm [7]. The study focused on the effect of PhF on implants, which was placed in different groups of the posterior maxilla according to CBCT (cone-beam computed tomography) grayscale for bone density [29]. The results showed that PhF could increase initial implant stability in posterior maxilla, thus allowing a faster loading protocol [29]. Another UV device, the TheraBeam SuperOsseo (Ushio Inc., Himeji, Japan) was used in a clinical trial from Germany, which used the implant removal torque value as an indirect reference of BIC in 360 implants of 180 patients [19]. The UV light of the TheraBeam SuperOsseo was delivered as a mixture of spectra; the intensity was 0.05 mW/cm^2^ at λ = 360 nm and 2 mW/cm^2^ at λ = 250 nm [34]. The finding from this research showed that PhF improved early-phase healing and stability and promoted the speed of osseointegration [19]. Shah reported that the pretreatment of dental implants with UV light revealed a statistically significant difference only in implant stability but not in other parameters, including mean marginal bone loss, pink/ white aesthetic score, and success/survival rate [21].

In the spine surgery of 13 patients, the result showed no significance in osteosclerosis between UV-treated and UV-untreated cages in lumbar fusion [22]. The ratio of the carbon attachment of titanium cages (20% at one year) in orthopedics was less than that in dental titanium instruments (60% at 4 weeks); thus, the effect of the UV photofunctionalization of titanium instrumentation in spine surgery was questionable [22]. However, UV-treatment could improv the osseointegration of aged 3D-printed porous Ti6Al4V scaffolds in the femur condyles of rabbits in a recent study [62]. It is possible that photofunctionalization has a positive effect in the further application of orthopedics.

## 5. Discussion

The mechanisms behind the enhanced osseointegration of dental implants after photofunctionalization are due to improving hydrophilicity and eliminating hydrocarbon contamination on the implant surface [25]. The UVA (wavelength range from 320 to 400 nm) and UVC (wavelength range from 200 to 280 nm) irradiation could result in hydrophilicity and the nano-scale modification of the titanium surface [25,65]. However, the vital mechanism behind excellent osseointegration might be because of carbon removal from the titanium surface by UVC [4,5]. In addition to antibacterial effects, UV activation would enhance the adsorption of plasma proteins of human body and improve osteogenic cell attachment, spreading, and proliferation [11,25,60]. Thus, it is possible to shorten the dental implant treatment time.

There is a conspicuous bacterial colonization on implants only 30 min after implant insertion [66,67], which may be prevented by UV-photofunctionalization restricting the growth of oral bacteria and biofilm [9,10,11]. Peri-implant-diseases-associated biofilms would affect the long-term outcome of dental implants. The microbial composition between periodontitis and peri-implantitis are similar; however, dental implants are more susceptible to oral infections due to anatomic and physiologic differences from natural teeth [67,68]. In addition, the peri-implant tissue response, including pro-inflammatory state, is influenced by transmucosal abutment geometry and surface [68]. Thus, the positive effect of photofunctionalization for the attachment of gingival fibroblasts or epithelial cells on implant abutment surface, which may decrease the severity of peri-implant infection [36,38,39,40].

There were some disadvantages in pre-clinical studies, which resulted in a risk of bias [23,24]. The quality assessment revealed that no animal study revealed a low risk of bias for all domains [23,24]. However, photofunctionalization still showed a benefit in the initial phase of osseointegration in different animal models [24]. The limitations in the clinical studies included differences in the age of patients, photofunctionalization protocol, experience of users, and follow-up period. Except for one German study, other studies were performed in Asia. The publication bias in the clinical studies would limit the significance of this contribution to implant dentistry. Thus, it is necessary to prove a positive effect in Western people through more studies. Photofunctionalization could overcome the challenge of complex dental implant cases, except for cancer-related cases with bone resection, segmental defect, or radiation [18]. Changing the photofunctionalization protocol (UV treatment for 15 min, then cleaning ozone for 5 min) may have an advantage in these complicated cases.

## 6. Conclusions

Many methods have been used to promote osseointegration for successful implant therapy, including photofunctionalization. UV photofunctionalization can change the surface wettability and eliminate the hydrocarbons that are generated by aging on the implant surface. Photofunctionalization can also enhance cell migration, attachment, and proliferation to promote osseointegration and coronal soft tissue seal. However, photofunctionalization did not overcome the cancer-related pathological condition and had little effect on the resistance to oblique forces. Moreover, the clinical assistance of photofunctionalization is still limited by the field of dental implants. To use the results, therefore, it is necessary to have more clinical trials focused on complex implant cases and non-dental fields in the future.

## Figures and Tables

**Table 1:** The effect of photofunctionalization on dental implants in vitro.

Author	Material	Method	Results with UV Treatment
To Reverse the Biological Aging/Degradation of Implant Surface			
Iwasa F et al. 2011 [6]	Ti with micro-nano-hybrid topography vs. Ti with microtopography alone Rat bone-marrow-derived osteoblasts	UV using a 15 W bacterial lamp for 48 h Stored for 8 weeks, then the PhF of a fresh, 3-day-old and 7-day-old UV-treated implant surface	Slower rate of the time-dependent degradation of titanium bioactivity after UV treatment in micro-nano-hybrid topography implant compared to that in microtopography alone
Tuna T et al. 2015 [7]	Zirconia-based discs Smooth vs. rough surfaces	UV using TheraBeam^®^ Affiny for 15 min	To decrease carbon by 43–81% To increase oxygen by 19–45% and zirconia by 9–41% To change from hydrophobic to hydrophilic No topographic change
Roy M et al. 2016 [26]	Commercial osteoplant base and rapid titanium dental implants	UVC with TheraBeam^®^ SuperOsseo for 12 min	To reverse the biological aging of titanium by reducing carbon contamination (up to 4-fold) To reduce surface H_2_O To increase TiOH with many -OH groups To improve biologic results No change in the topography of the surface
Roy M et al. 2017 [27]	ZrO_2_ (Zr similar to titanium)	UVC with TheraBeam^®^ SuperOsseo for 12 min	To reduce carbon 3-fold No change in crystalline structure
Arroyo-Lamas N et al. 2020 [28]	Ti with Ti oxide surface	UVC for 12 min Mercury (Hg)-vapor (λ = 254 nm) vs. Light-emitting diodes (LEDs; λ = 278 nm)	To reduce the concentration of surface hydrocarbons (26~23.4 C at %) To increase the concentration of O_2_ and Ti Hg-vapor lamps could be replaced by LED-based technology
Roy M et al. 2021 [29]	TiO_2_, ZrO_2_, polyether-ether-ketone (PEEK)	UVC using TheraBeam^®^ SuperOsseo for 12 min	To remove hydrocarbons (twofold in PEEK; threefold in TiO_2_ and ZrO_2_) To decrease the harmful effect of the biological aging of the implant surface
Jaikumar RA et al. 2021 [30]	Zirconia implant	UV (λ = 254 nm) for 48 h	UV group vs. control group: Oxygen concentration: 42.8% vs. 29.09% carbon concentration: 34.34% vs. 45.41% To enhance the surface topography and hydrophilicity
To decrease peri-implant stress distribution			
Ohyama T et al. 2013 [31]	3-dimensional finite element analysis of different lengths with various BICs (53.0% and 98.2%)	BIC 98.2% as UV treatment	To diminish peri-implant stress by 50% (BIC 98.2% vs. 53.0%); 15% (implant length from 7 to 13 mm) To improve the effective distribution of peri-implant stress rather than implant length Similar results under oblique load
Ohyama T et al. 2017 [32]	Unique finite element analysis model with a 3D model (BIC: 53.0% or 98.2%)	BIC 98.2% as UV treatment Vertical or oblique loading	To reduce stress on surrounding tissues UV treatment vs. wider implants: Greater effect in vertical loading Less effect in oblique loading
Antimicrobial effect			
Yamada Y et al. 2014 [9]	Wound pathogens such as Streptococcus pyogenes and Staphylococcus aureus Titanium disc	UVA (λ = 352 +/− 20 nm) or UVC (λ = 254 +/− 20 nm) with a mercury lamp for 48 h (500 J/cm^2^)	To decrease the amount of bacterial attachment and accumulation To change wettability, as per the modification to a super hydrophilic surface To reduce carbon content (UV-C better than UV-A in the above items) No change in topography
De Avila ED et al. 2015 [10]	Titanium disc Oral microbial community	UVC using TheraBeam^®^ SuperOsseo for 12 min	To create and maintain an antimicrobial surface To change the bacterial community profile To reduce bacterial attachment and biofilm formation
Jain S et al. 2018 [11]	Streptococcus sanguinis Anodized layer with a anatase phase Anodized layer with anatase and rutile phases	15 W UVA (Philips; λ = 365 nm) or 15 W UVC (Philips; λ = 254 nm) for 10 min	To reduce bacterial attachment by at least 20% Achieve at least 50% killing efficacy by UVA in an anodized layer with an anatase phase Achieve at least 50% killing efficacy by UVA or UVC in an anodized layer with anatase and rutile phases.
Different cell studies			
Shen JW et al. 2016 [33]	MC3T3-E1 cells Aqueous medium (dH_2_O)	UV using a 15 W bacterial lamp for 24 h 5 groups of Ti implants (SLAnew, SLAold, modSLA, UV-SLA and UV-modSLA)	To remove hydrocarbon contamination on titanium stored in air or water To increase cell attachment, proliferation, ALP activity, and osteocalcin release
Henningsen A et al. 2018 [34]	Murine osteoblasts Sandblasted and acid-etched titanium discs	UVC using TheraBeam^®^ SuperOsseo for 12 min	To increase the oxidation of the surface To decrease the carbon on the surface No change in surface structure To promote osteoblast attachment and growth.
Ikeda T et al. 2021 [35]	Osteoblasts derived from rat bone marrow Acid-etched titanium disks	UV treatment	To decrease bioactivity when the temperature fluctuates by ≥20 °C above or below room temperature (25 °C) (particularly toward lower temperatures), independent of the hydrophilicity/ hydrophobicity To restore temperature-compromised bioactivity using UV treatment
Mehl C et al. 2017 [36]	Gingival fibroblast Abutments: zirconium dioxide and titanium alloy	UVC using TheraBeam^®^ SuperOsseo for 12 min Argon plasma Ultrasound disinfection	To increase cell adhesion on zirconium dioxide by UV, argon plasma, or ultrasound disinfection To increase cell adhesion on a titanium alloy with ultrasound disinfection
Harder S et al. 2019 [37]	Human whole blood Titanium with SLA surface	UVC using TheraBeam^®^ SuperOsseo for 12 min	To suppress the gene expression of IL 1β for 1–8 h (TNF gene not significantly altered).
Nakhaei K et al. 2020 [38]	Human epithelial cells Pure titanium discs	UVC using TheraBeam^®^ SuperOsseo for 12 min	To remove carbon contamination by reducing C-C and C = O groups To enhance the attachment, adhesion, and retention of epithelial cells on implants
Okubo T et al. 2020 [39]	Human epithelial cells Titanium discs with a machined or polished surface	UVC using TheraBeam SuperOsseo for 12 min	To remove the chemical contamination of the polished surface To increase the number of attached epithelial cells on the implant To increase the number of adherent cells after mechanical detachment
Razali M et al. 2021 [40]	Human gingival keratinocytes and fibroblasts Yttria-stabilized zirconia, alumina-toughened zirconia, and pure titanium abutments	UVC using Therabeam^®^ SuperOsseo for 12 min.	To improve the biological seal of the surrounding soft tissue peri-implant interface Yttria-stabilized zirconia with the best biological seal among these materials

ALP: alkaline phosphatase; BIC: bone-to-implant contact; SLA: sandblasted acid-etched; Ti: titanium; UV: ultraviolet; UVA: ultraviolet A; UVC: ultraviolet C; 3D: three dimensions. PEEK: please to see material (polyether-ether-ketone). LEDs: Light-emitting diodes.

**Table 2:** Preclinical animal studies of photofunctionization.

Author	Material	UV Light	Results with UV Treatment
Rat model			
Aita H et al. 2009 [5]	Machined and acid-etched Ti In 9 rats (8 weeks old)	UVA/UVC for variable time up to 48 h	To enhance osteoconductive capacity To accelerate implant fixation 4-fold
Ikeda T et al. 2014 [53]	Nanofeatured Ti Femurs of 6 rats (8 weeks old)	UV-T for 15 min using TheraBeam Affiny	To improve the strength of osseointegration by a push-in test (2.2-fold in week 2 and 2.3-fold in week 4 of healing)
Sugita Y et al. 2014 [54]	Ti femurs of 10 genetically modified rats (phenotype close to human type 2 diabetes; 10 weeks old)	UV for 15 min using TheraBeam Affiny	To increase the strength of osseointegration (1.8-fold in week 2 and 3-fold in week 4 of healing) in a rat model of type 2 diabetes
Minamikawa H et al. 2014 [8]	Ti_6_Al_4_V (smooth or rough surface) Femurs of 6 rats (8 weeks old)	UV-T for 15 min using TheraBeam Affiny	To convert the Ti_6_Al_4_V surface from hydrophobic to super-hydrophilic (however, the conversion to hydrophobic takes 4 weeks) To improve the strength of the bone–implant integration of both surfaces (UV treatment on a smooth surface > no treatment on a rough surface)
Tabuchi M et al. 2015 [57]	Ti-6Al-4V mini-screw Femurs of 6 rats (8 weeks old)	UV for 12 min using TheraBeam SuperOsseo device	To change from hydrophobic to super-hydrophilic To increase resistance against the tipping force by 1.5~1.7-fold To gain a strong elemental peak of calcium and phosphorus
Tabuchi M et al. 2015 [58]	Ti-6Al-4V mini-screw Femurs of 6 rats (8 weeks old)	UV for 12 min using TheraBeam SuperOsseo	To improve anchoring capability Less displacement under lateral tipping force More intact and contiguous regenerated bone tissue
Brezavscek M et al. 2016 [43]	Zirconia-based disc Femurs of 88 rats (8 weeks old)	Push-in test (48 rats) UV for 15 min using TheraBeam Affiny	To increase push-in values at Week 2 by 2.1–2.8-fold and at Week 4 by 1.7–2.0 fold To increase the BIC on a smooth surface by 3~7-fold and on a rough surface by 1.4~1.7-fold To enhance the strength of the bone–implant interface by 2-fold (40 rats)
Ishijima M et al. 2016 [55]	Ti mini-implants Femur of 6 aged rats (15 months old)	UV for 12 min using TheraBeam SuperOsseo	To enhance the strength of osseointegration by 40% in aged rats Strong elemental peaks of calcium and phosphorus
Hirota M et al. 2017 [44]	Acid-etched Ti implants (1 × 4 mm) and Ti mesh Femurs of 20 rats (8 weeks old)	Half of implants in 2 mm defect, half exposed UV for 12 min using TheraBeam SuperOsseo	To enhance vertical ridge augmentation and bone–implant contact To increase the strength of osseointegration (3-fold) To enhance the closure of the bone–implant gap
Soltanzadeh P et al. 2017 [56]	Ti implants Femurs of 7 rats (8 weeks old)	0.46 N of constant lateral force UV for 12 min using TheraBeam SuperOsseo	To increase the success rate (100% vs. 28.6%) To increase the strength of osseointegration (2.4-fold) To decrease the implant title degrees (0.5-fold)
Taniyama T et al. 2020 [41]	Ti implants (1 × 2 mm) Femurs of sham-operated vs. ovariectomized rats (each *n* = 8; 12 weeks old)	Rat osteoporosis model Push-in test UV for 12 min using TheraBeam SuperOsseo	Titanium with vs. without UV treatment: Contact angle of H_2_O: </=5 degrees vs. >/=80 degrees To enhance bone–implant integration in ovariectomized rats (80% higher than control titanium) To increase the push-in value in both groups by 50–70%
Rabbit model			
Sawase T et al. 2008 [1]	Ti implant with the anatase form of a TiO_2_ surface Tibia of 6 rabbits (28–36 weeks old)	UV for 24 h	To improve initial cell reactions To enhance early bone formation by increasing BIC
Jimbo R et al. 2011 [60]	Fluoride-modified TiUnite implants Tibial metaphyses of 12 rabbits (28–36 weeks old)	UV (352 nm) for 24 h	To enhance BIC and bone apposition during early stages of osseointegration (2 and 6 weeks)
Hayashi M et al. 2014 [52]	TiO_2_ powder spin-coated onto pure titanium disc tibiae of 9 rabbits	UV (352 nm, 6 W) for 24 h	To upregulate gene expression (ALP, RUNX-2, and IL-10) To continue the biologically enhancing effect even after 12 weeks of healing time
Yamazaki M et al. 2015 [61]	Acid-etched pure titanium screws Femur of 20 rabbits (16 weeks old)	UVC (3 mW/cm^2^) for 48 h using a 15 W bactericidal UV bench lamp (254 nm)	To gain a higher density of cells, as well as thicker and longer bone tissue attachments To increase the volume of cortical-like tissue in the coronal region
Shen J et al. 2016 [42]	Ti implants (4 × 8 mm) Tibial metaphyses and femoral condyles of 40 rabbits	32 implants × 5 groups: SLAnew, SLAold, modSLA, UV-SLA, and UV-modSLA UVA/UVC for 24 h	To eliminate hydrocarbon contamination To enhance bone-to-implant contact (interfacial strength) and osseointegration
Kim HS et al. 2017 [49]	Commercial Ti implants (4 × 6 mm) Tibia of 12 rabbits (>12 weeks old)	ALN on titanium surface UV at 189.4 nm and 253.7 nm of wavelength for 2 h using UV-Cleaner	To increase per-implant bone formation and osseointegration Highest bone–implant contact in the UV+/ALN+ group
Lee JB et al. 2019 [50]	Machined SLA surface Ti implants Tibia of 4 rabbits (12 and 16 weeks old)	UVC for 48 h	Higher bone-to-implant contact ratio at 10 days To gain earlier osseointegration in a machined surface implant after UV treatment than in an SLA surface implant
Sanchez-Perez A et al. 2020 [46]	20 commercial Titanium implants (3.75 × 8 mm) 5 rabbits (3–3.5 kgs)	UVC-lamp (254 nm; 6 W) at a distance of 15 cm for 15 min	Did not improve the percentage of BIC at 8 weeks More homogenous BIC values in the UV group
Yin C et al. 2022 [62]	3D-printed porous Ti_6_Al_4_V scaffolds in a dark place for 4 weeks Bilateral femur condyles of 27 mature male New Zealand rabbits	A irradiation cube: >2 MW/cm^2^ (270 nm) and 30 MW/cm^2^ (365 nm) for 15 min	To enhance hydrophilicity, cytocompatibility, and alkaline phosphatase activity, while preserving their original mechanical properties in vitro To promote bone ingrowth, the bone–implant contact ratio, and the mineralized/osteoid bone ratio in vivo
Dog model			
Hirakawa Y et al. 2013 [48]	Ti implants with TiO_2_ surface Mandible of 6 beagle dogs	Plasma source ion implantation method UVA (352 nm) for 24 h	To improve serum fibronectin attachment To increase BIC after 2 weeks healing (42.7% vs. 28.4% in control) To accelerate early osseointegration by a combination of plasma fibronectin and plasma source ion implantation
Pyo SW et al. 2013 [47]	Commercial Ti implants Both jaws of 4 dogs (72–96 weeks old)	UV for 15 min using TheraBeam Affiny	To convert an implant surface from hydrophobic to super-hydrophilic To increase removal torque by 50% and BIC To promote interfacial bone deposition and marginal bone seal
Kim MY et al. 2016 [63]	Ti implants Mandibular premolars of 4 female beagle dogs (24 weeks old)	2 as control vs. 2 as UV UV for 15 min using TheraBeam Affiny	To gain better osseointegration To increase bone-to-implant contact (BIC) and new bone formation
Huang Y et al. 2022 [59]	Aged Ti-implant Mandibular premolars of 8 male beagle dogs	12 as control UVC for 1/6 h (12) UVC for 1/2 h (12) UVC for 1 h (12)	There were significantly higher BV/TV and bone–implant contact at 4 weeks; however, there were no significant differences at 12 weeks. The effect was independent on the UV-C duration.
Minipig model			
Mehl C et al. 2018 [51]	48 titanium implants Both jaws of 3 mini-pigs	UVC for 48 h	To attain higher earlier osseointegration No significant differences in bone–implant contact (BIC) and implant stability quotient (ISQ) in 9 months

ALN: alendronate; BIC: bone-to implant contact; BV/TV: trabecular bone volume to total volume fraction; IL-10: interleukin-10; ISQ: implant stability quotients; RUNX-2: runt-related transcription factors 2; Ti: titanium; UV: ultraviolet; UVA: ultraviolet A; UVC: ultraviolet C.

**Table 3:** Clinical studies of dental implant therapy using photofunctionization.

Author	Study Type	Material and Method	Results with UV Treatment
I-1. Dentistry			
Ogawa T. Study Group (Japan)			
Funato A et al. 2013 [12]	Case series	7 implants (3i Biomet, Certain) in the compromised bone of four patients UV machine (TheraBeam Affiny; Ushio Inc., Tokyo, Japan): UV treatment for 15 min	Complex cases: fresh extraction socket, sinus elevation, vertical ridge augmentation, and the immediate replacement of failing implant ISQs from 48–75 at placement to 68–81 at loading To gain more ISQ between implant placement and loading in cases with lower primary stability To increase or to maintain marginal bone level at one year after loading ⇨ PhF enhanced OSI (increased ISQ per month) in complex cases ⇨ PhF shortened osseointegration time
Funato A et al. 2013 [13]	Retrospective study	168 implants in 70 patients (with UV treatment) vs. 222 implants in 95 patients (without UV treatment) UV machine (TheraBeam Affiny; Ushio Inc. Tokyo, Japan): UV treatment for 15 min	To shorten healing time before loading: 3.2 months vs. 6.5 months To promote “OSI” in different primary stability subgroups: “2.0–8.7” vs.”1.8–2.8” Similar implant survival rate: 97.6% vs. 96.3% ⇨ PhF allowed for a faster loading protocol without compromising the implant success rate
Suzuki S et al. 2013 [14]	Prospective study	33 implants (NobleReplace, TiUnite) in the maxilla of 7 patients UV machine (TheraBeam Affiny; Ushio Inc. Tokyo, Japan): UV treatment for 15 min	To increase ISQ in UV treatment groups: eliminating stability dip, 78.0 at 6 weeks vs. 66.1 at 2~6 months in “literature as-received implants” To promote OSI in UV treatment groups: 6.3 in “initial ISQ from 65 to 70” and 3.1 in “initial ISQ from 70 to 75” vs. −3.0 to 1.17(average: -0.10) in “literature as-received implants” ⇨ PhF accelerated and enhanced the osseointegration of dental implants
Kitajima H et al. 2016 [15]	Retrospective study	55 implants (3i Biomet, Certain) in 38 patients with ISQs < 60 at placement UV machine (TheraBeam Affiny; Ushio Inc. Tokyo, Japan): UV treatment for 15 min	190.9% implants in complex cases: GBR, sinus lift or fresh extraction sockets; 9.1% in regular cases Implant success rate: 98.2% after 2–3 years follow-up To increase in ISQs from 50.4 +/− 7.7 at placement to 74.3 +/− 5.7 at Stage II surgery; average healing time 7.1 +/− 2.1 months OSI (OSI as “ISQ at Stage II–ISQ at placement/healing time”) of low initial-stability implants (ISQs < 55): 3.9–4.7 in the UV group vs. 0.36–2.8 in the as-received group ⇨ PhF was more effective for implants with lower primary stability
Hirota M et al. 2016 [16]	Retrospective case-control study	49 implants (Branemark MKIII TiUnite) in 7 patients; 24 as-received and 25 with UV treatment UV machine (TheraBeam Affiny; Ushio Inc. Tokyo, Japan): UV treatment for 15 min	Complex cases: GBR, sinus lift, fresh extraction sockets To accelerate OSI both for regular and complex cases, especially more pronounced in cases with poor quality bone and complex cases To increase final ISQ at Stage II in the UV group regardless of primary stability and innate bone support at implant placement ⇨ PhF was a stronger determinant of implant stability than other factors
Hirota M et al. 2018 [17]	Retrospective study	563 implants in 219 patients Bone quality classification with CT Hounsfield unit (HU): D1~D4 UV machine (TheraBeam Affiny; Ushio Inc., Tokyo, Japan): UV treatment for 15 min	D1: >1250 HU; D2: 750–1250 HU; D3: 375–750 HU; D4: 150–375 HU Early implant failure rate: subjects with UV treatment vs. subjects without UV treatment: 1.3% vs. 4.3% ⇨ To reduce the early implant failure rate after UV treatment
Hirota M et al. 2020 [18]	Prospective study	70 implants in 16 patients for follow-up after 7 years, including regular cases, complex cases, and cancer-related patients Bone quality classification with CT Hounsfield unit (HU): D1~D5 CT, bone quantity: A~E UV machine (TheraBeam Affiny; Ushio Inc. Tokyo, Japan): UV treatment for 15 min, then cleaning ozone for 5 min	Regular (no site development or cancer): 30 implants into analysis + 4 implants in sleep; Complex (GBR, sinus lift, fresh extraction sockets): 21 implants; Cancer (cancer-related resection +/− radiation): 15 implants Success rate in regular cases, complex cases, and cancer-related patients: 100%, 100% and 22.2% ⇨ Did not overcome the challenges of a pathophysiologically compromised oral condition
I-2. Other study groups			
Puisys A et al. 2020 (German) [19]	Triple-blinded, split-mouth, randomized controlled clinical trial	360 implants in 180 patients; 180 (UV; 71 in maxilla, 109 in mandible) vs. 180 (control; 71 in maxilla, 109 in mandible) 2. Placement; Groups 1~6 (weeks 1, 2, 3, 4, 6, and 8) 3. UV device (TheraBeam SuperOsseo; Ushio Inc., Sazuchi Bessho-cho, Himejij, Hyogo, Japan): for 12 min 4. wavelength: 180–300 nm	To increase the RT value (indirect information of BIC) Significant difference in the RT value between UV and control: in groups 2, 3, 4, and 6. ⇨ To improve healing and implant stability, especially in the early phase ⇨ To increase the speed of osseointegration
Choi B et al. 2021 (Korea) [20]	Parallel-designed randomized double-blinded clinical trial	57 implants in the posterior maxilla of 34 patients; 29 (UV) vs. 28 (control) CBCT grayscale value: >500: bone quality group II; 300~500: group III <300: group IV UV machine (TheraBeam Affiny; Ushio Inc. Tokyo, Japan): for 15 min	Group III: significant difference in ISQ at 4 weeks and 4 months Group II: significantly less bone loss in the UV-treatment group at 4 weeks Others: no significant difference between the UV-treatment and the control group ⇨ To increase initial stability in posterior maxilla with poor bone density ⇨ To allow a faster loading protocol
Shah SA et al. 2021 (India) [21]	Randomized controlled trial	Immediate implants in the anterior maxilla of 90 patients Control group and implants pretreated with platelet-rich plasma (PRP group) or photofunctionalization (PF group) UV radiation of wavelength 253.7 nm in an ultraviolet ray chamber (SK Dent) for 20 min	Pretreatment with PF or PRP: statistically significant difference only in implant stability but not in other parameters (including marginal bone loss, pink/white aesthetic score and success/survival rate)
II. Orthopedics			
Tominaga H et al. 2019 [22]	Prospective study	13 patients underwent lumbar fusion Prospace intervertebral cage (B-Braun Company, Germany) UV using a low-pressure mercury lamp (TheraBeam Affiny; Ushio Inc. Himeji, Japan): for 15 min wavelength of 254 nm and 9.5 mW/cm^2^	To change the surface hydrophilic from hydrophobic To decrease the amount of carbon attached on the cage No significant difference between the degree of osteosclerosis between titanium cages with UV and without UV treatment ⇨ Note: UV photofunctionalization in spine surgery is questionable.

BIC: bone-to-implant contact; CBCT: cone-beam computed tomography; CT: computed tomography; ISQ: implant stability quotients; HU: Hounsfield unit; OSI, osseointegration speed index; PhF, photofunctionalization; RT, removal torque; UV, ultraviolet.
